# Vibroacoustic analysis of dental air turbine noise

**DOI:** 10.1038/s41405-022-00117-5

**Published:** 2022-09-10

**Authors:** Wonsup Lee, Ho-Beom Kwon

**Affiliations:** 1 Clinical Assistant Professor, Department of Prosthodontics, Dental Hospital, St. Mary’s Hospital of Catholic University of Korea, Seoul, 06591 Republic of Korea; 2 Professor, Dental Research Institute and Department of Prosthodontics, School of Dentistry, Seoul National University, 101, Daehak-ro, Jongno-gu, Seoul, 03080 Republic of Korea

**Keywords:** Dental equipment, Occupational health

## Abstract

**Objective:**

The objective of this study is to analyze the noise mechanism of dental air turbine handpiece with vibroacoustic simulation.

**Materials and methods:**

The operational part of the Gentle Silence Lux 8000B (KaVo Dental GmbH) was disassembled and scanned. The scanned data were rendered to smooth irregularities and then virtually reassembled. The rendering was 3D mesh modeled for the analysis. And, the interior void space and exterior space was mesh modeled as air layer. As per simulation input informations, the material property of steel was provided for the handpiece components. Supplied air pressure of 0.22 MPa at the inlet and static temperature of 25 °C was provided as operating conditions. Twenty virtual microphones were arrayed to measure the noise. Vibroacoustic noise simulation was performed with ACTRAN 2021 (MSC software corporation).

**Results:**

The mean value of noise ranged from 49.88 to 66.38 dB while the peak value ranged from 69.53 to 81.64 dB depending on the microphone position. All microphones showed the similar noise pattern which had peak amplitude at around 4500 Hz. The calculated natural frequency of interior air layer was 4478.92 Hz and 7573.77 Hz.

**Discussion:**

The simulated result showed similar tonal noise of dental handpiece suggesting air resonance phenomenon as a possible cause of dental handpiece noise.

**Conclusion:**

Vibroacoustic analysis of the air layer contained within the dental air turbine handpiece showed the resonance peak noise at 4478.92 Hz under the simulated conditions.

## Introduction

Awareness of the harmful effects of dental air turbine noise has appeared in the literature since the late 50 s [1–5]. Concerns have been raised about hearing impairment to dentist from dental air turbine noise, and there have been numerous articles reporting an association between dental air turbine noise and hearing damage [6–9]. After reviewing 17 articles that assessed noise level in dental environments, Henneberry et al. warned oral health professionals of potential hearing loss due to exposure to excessive noise limits (85 dBA) [10]. It has also been reported that the noise of a dental air turbine can cause fear and anxiety in patients [10–14]. Dental air turbine noise can create a hazardous environment for both dentists and patients [15–19]. While there are contrary reports regarding the possibility of hearing damage due to dental air turbine noise [20–24], it may be quite agreeable that the dental air turbine noise is unpleasant in every sense.

Altinoz et al. analyzed the peak noise frequency band of a dental air turbine [25]. The author analyzed the frequency bands of noise emitted from five types of air turbines under eight different working conditions. The noise was recorded with a microphone located 30 cm from the handpiece. The measured frequency of the peak noise ranged from 4638 to 11988 Hz with the average of 6960 Hz. It appeared that the noise amplitude pattern was not even throughout the entire audible frequency band, but rather, there was a peak frequency that creates typical sharp handpiece tonal noise. It is unclear how this tonal noise was being generated.

Kusano et al. replaced the case of a dental handpiece from metal to plastic and evaluated the noise level [26]. Besides the outer casing, the rest of the parts such as air turbines, pipes and bearings were still metal. The noise decreased to some extent, but not significantly. Disposable handpieces were evaluated by the American Dental Association Council on Scientific Affairs [27]. The examined noise level was similar to the conventional air driven handpiece. This was in accordance with the other report according to Dyson [28]. The noise characteristics of air turbine handpiece was not affected by the material of the handpiece. These results might suggest that the handpiece noise is caused by air borne noise since the structure borne noise, the noise generated and transmitted through the vibration of the structure, should have been affected by the material of the structure.

Provided that the handpiece noise is air borne noise, there is a possibility that the handpiece tonal noise is vibroacoustic noise which is induced by resonance of the air. However, it seems the information is lacking to identify this assumption. The purpose of this study is to analyze the noise generation mechanism of dental air turbine handpiece in vibroacoustic aspect.

## Materials and methods

**Fig. 1:**
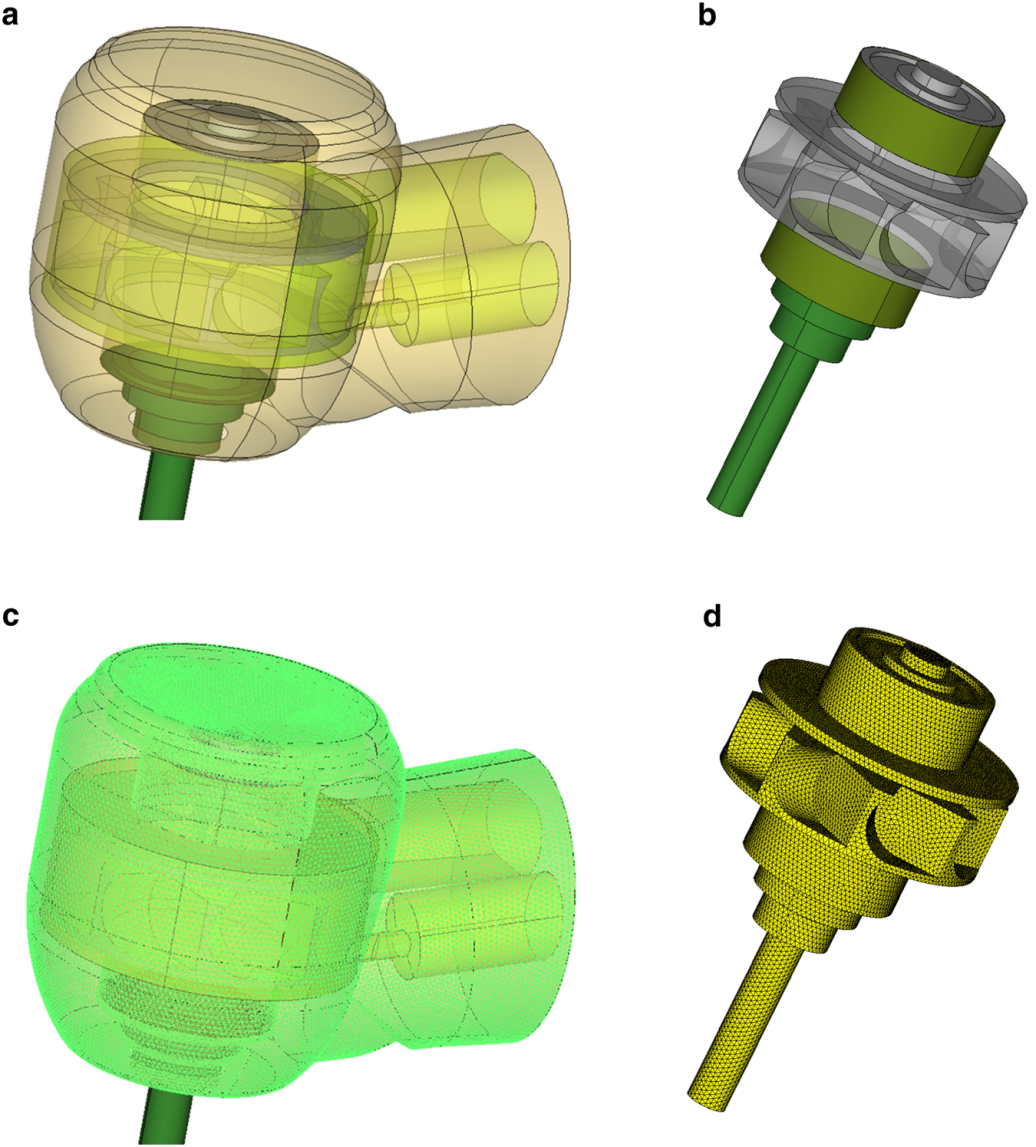
3-D modelling process. **a**, **b** Reassembled components after rendering. **c**, **d** Mesh models converted from renderings.

The operational part of the Gentle Silence Lux 8000B (KaVo Dental GmbH), where the air turbine is contained, was disassembled and scanned with Handyscan black Elite (Ametek, Inc.) to create a 3-dimensional model for the analysis. To overcome the measurement resolution problem, the scanned data of the disassembled components were rendered to create smooth surface and remove any irregularities. The rendered separate domains were then virtually reassembled. The reassembled renderings were converted to a mesh model for the analysis (Fig. 1).

**Fig. 2:**
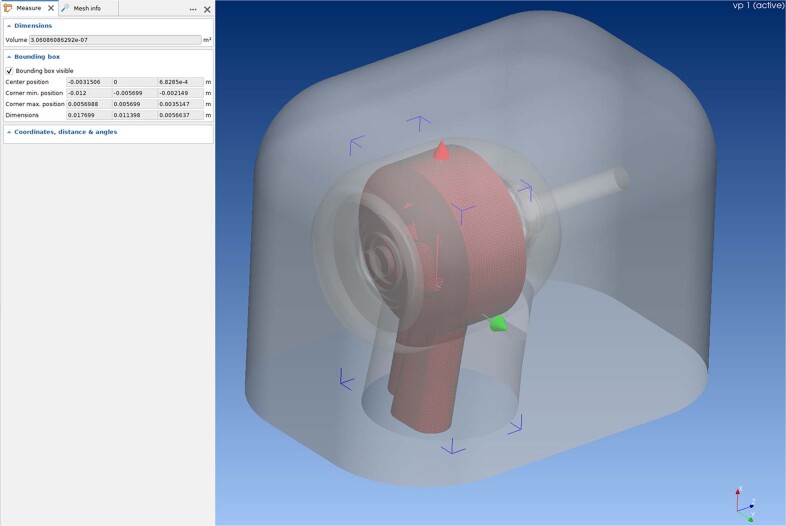
The air layer mesh model within the handpiece and the surrounding exterior space. The air layer within the handpiece is depicted as brown color. The exterior space air layer is depicted as grey color. X, Y, and Z axis are shown as red, green, and blue arrows.

The coordinate system was determined as shown in Fig. 2. And the interior void space was mesh modeled as air layer, as well exterior air space surrounding the handpiece (Fig. 2). The dimension of the exterior air space was 22.4 × 20.6 × 28.6.

Material properties that were used in this simulation are provided in Table 1. As there was no information regarding the metal the handpiece was made of, the property of steel was used arbitrarily. Operating conditions were: supplied air pressure of 0.22 MPa at the inlet and static temperature of 25 °C. The inlet air velocity was arbitrarily set to 100 m/s. The number of elements is given in Table 2.

**Table 1:** Material properties.

Air
Density: 1.225 Kg/m^3^
Sound Speed: 340 m/s
Steel
Density: 7800 Kg/m^3^
Poisson Ratio: 0.31
Young Modulus: 210 GPa

**Table 2:** Number of elements.

Acoustic mesh	223005
Structure mesh	645042
Near field acoustic mesh	1395650
Total	2263697

**Fig. 3:**
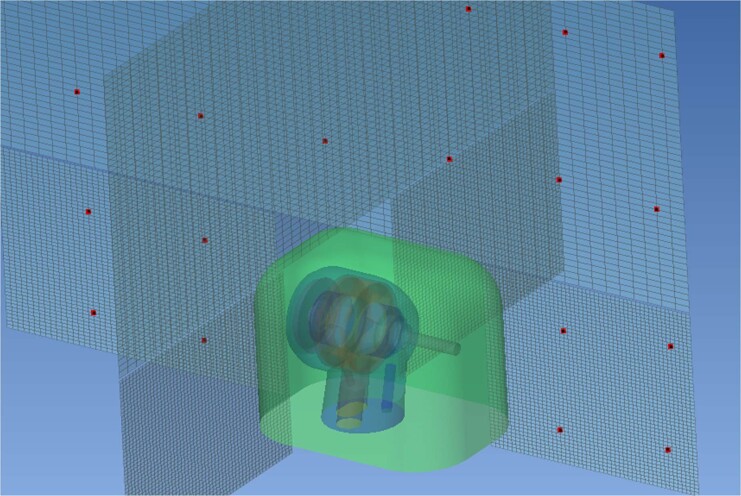
Microphone array. The positions of the virtual microphones shown as red dots.

**Fig. 4:**
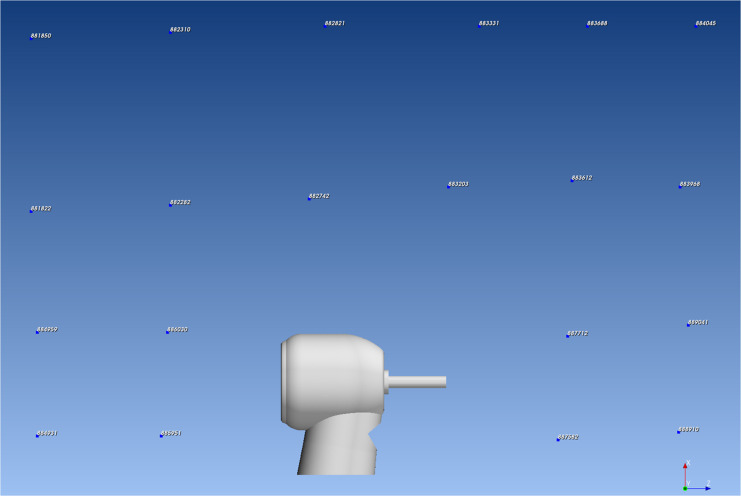
Microphone designation. The virtual microphones were designated as individual number.

The virtual microphones were arrayed in the XZ plane in a grid pattern (Fig. 3). In total, 20 virtual microphones were positioned (Fig. 4).

Modal analysis of the air layer contained within the handpiece head part was conducted. Frequency response analysis of air layer inside the handpiece to predict vibrational and acoustic characteristics was performed under the given condition; excitation from the compressed air inflow. The excitation frequency was provided from 1500 Hz to 10,000 Hz with the increments of 500 Hz. And noise radiation simulation surrounding the handpiece operational part were also conducted with the measured range from 1500 Hz to 10,000 Hz. These simulations were performed using the ACTRAN 2021 (MSC software corporation) with ACTRAN Vibro-Acoustics, ACTRAN Acoustics, and ACTRAN VI/PlotViewer modules.

## Results

The measurements from the microphone arrays regarding the loudness of the noise are shown in Table 3. The mean value of noise from the microphones ranged from 49.88 to 66.38 dB depending on the microphone position. The peak value ranged from 69.53 to 81.64 dB.

**Table 3:** The microphone measurements.

Microphone	Mean Value (dB)	Peak Value (dB)
881850	53.81	67.78
882310	57.43	71.82
882821	59.71	74.53
883331	58.71	73.68
883688	56.66	71.71
884045	54.36	69.53
881822	52.09	67065
882282	60.06	74.72
882742	66.38	81.64
883203	63.54	78.84
883612	58.57	74.00
883968	54.94	70.70
884959	49.88	70.71
886030	54.5	74.28
887712	57.27	74.18
889041	53.64	70.49
884931	57.12	74.68
885951	62.04	79.55
887582	57.13	74.00
888910	53.68	70.63

**Fig. 5:**
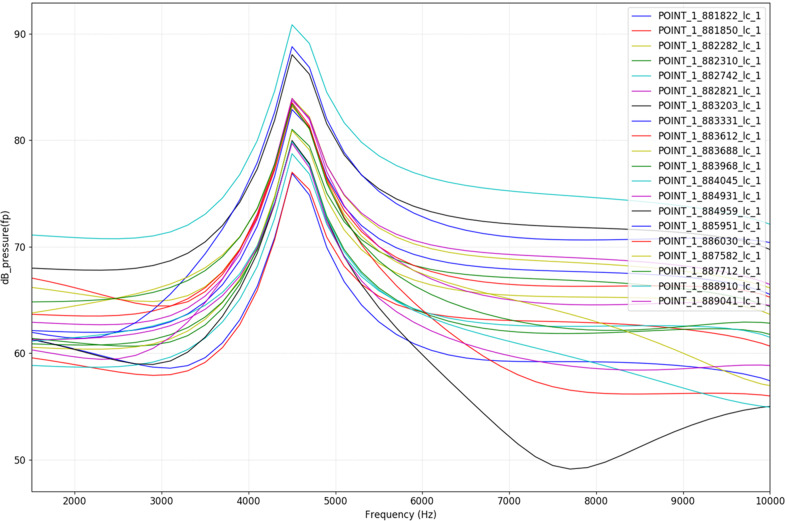
Sound measurements from the microphone array (dB). The measurements for each microphone are displayed in different color.

**Fig. 6:**
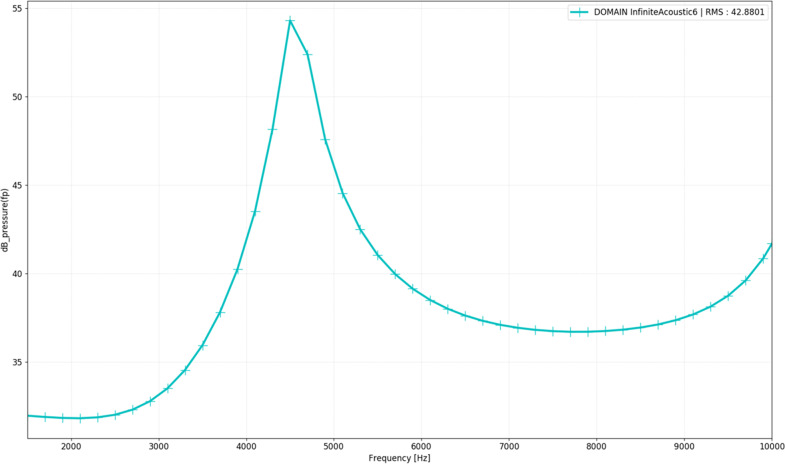
The radiated sound power (dB). The radiated sound power for the measured frequency is shown.

The measurements from the microphones arrays regarding the peak amplitude by frequency are shown in Fig. 5. The results showed that the individual noise patterns were similar with the peak amplitude at around 4500 Hz. The radiated sound power also showed similar result compared to the individual results (Fig. 6).

**Fig. 7:**
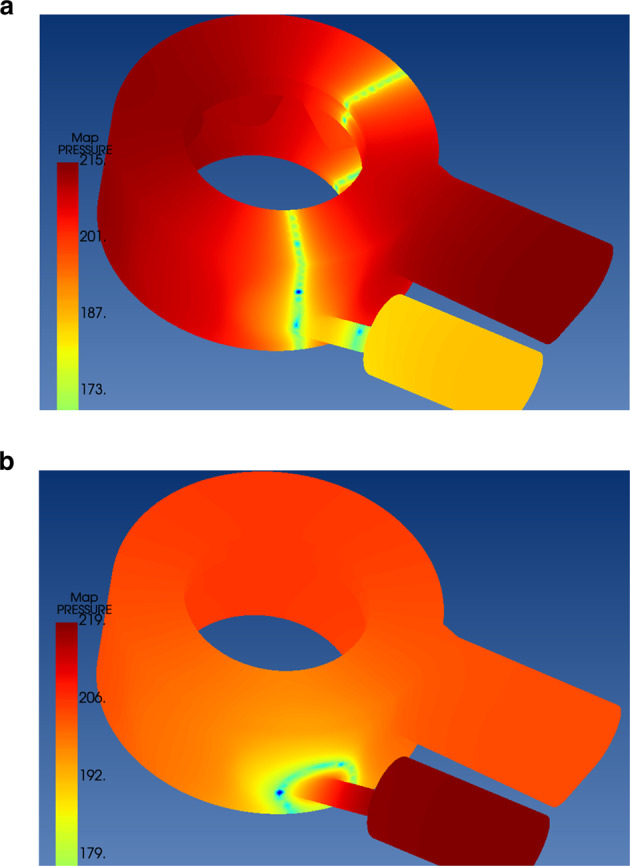
Visualized mode shape calculation results of interior air layer. **a** first mode and (**b**), second mode.

The calculated natural frequency of first mode was 4478.92 Hz, and the second mode was 7573.77 Hz. (Fig. 7)

## Discussion

The types of noise estimated to be generated by the dental air turbine handpiece are as follows: structure borne noise, blade passage noise, and air borne noise.

Structure borne noise, which is induced and transmitted via structural vibration, is unlikely to be the source of the tonal peak noise since there was no damping effect such as noise reduction by grabbing the handpiece [29]. According to Rogers, lubricating the bearings or polishing the internal surface of dental handpiece had no effect on noise reduction [30]. This result further suggest that vibration may not be the cause of the dental handpiece noise.

Blade passage noise can be caused by air interaction with the blades of the handpiece impeller. Blade passage frequency can be calculated with the following formula:

$$f_B = \frac{{rpm \times N}}{{60}}$$

where rpm stands for rotation per minute and N stands for the number of the blades [31]. Provided that the handpiece rpm is 350,000, the blade passage noise is 46,667 Hz which is beyond audible range.

Air borne noise can be either aeroacoustic noise or vibroacoustic noise. Aeroacoustic noise is induced by the flow of the air. Juraeva et al. analyzed the noise level of the dental air turbine handpiece with different design modifications using computational fluid dynamics software [32]. Their design modifications resulted in reduced noise level but it is unclear whether the tonal noise pattern was changed.

Vibroacoustic noise can be induced by air resonance. Resonance phenomenon of an enclosed air by acoustic or vibrational excitation has been studied in various fields [33–37]. The excitation source can be turbulent air inflow or vibration transmitted through casing [38–40]. This study analyzed the resonance phenomenon of air layer inside the dental handpiece excited by the inflow of compressed air. Vibroacoustic modal analysis which involves the determination of natural frequencies and associated vibration mode shapes was used. Resonance occurs when constructive interference occurs between the supplied air and the air layer inside the handpiece. The result of this study showed an audible resonance peak at 4478.92 Hz which can be identified as the tonal noise familiar to all dentists: typical dental air turbine handpiece noise.

The second natural frequency calculated from the theoretical modal analysis was not identified in virtual microphones. It is estimated that the second mode excitation did not emanate sufficient sound level to be detected by the virtual microphones due to the dipole pattern of the second mode shape. As shown in Fig. 7b, second mode appears to be a dipole source which could have an offset effect on each other yielding little or no net displacement to propagate beyond the medium. This assumption raises the need for complemenary experiments to refine and validate the results. However, There are some technical difficulties in validating the results. First, the object to be tested is a mass of air, not a structure which makes experimental or operational modal analysis rather difficult to perform. Second, the dynamics of air mass casts another difficulty in assigning even physical properties over every pixels. Also, the changing form of the air mass can further complicate the calculation. And lastly, the object is relatively too small compared to other frequently tested industrial experimental objects. These difficulties might have been a major obstacle to study the dental handpiece turbine noise with simulation methodologies. Further research shall be required to overcome the technical difficulties and to validate the claim that has been made by this study.

One technical limitation of this study was that the provided material property and boundary conditions might be different from the actual material property consisting the handpiece components as well as real boundary conditions. Another limitation was that the result of this study was solely based on vibroacoustic analysis, and therefore, it does not reflect other possible mechanism of noise generation such as aeroacoustic noise. However, despite these limitations, the results of this study suggest that resonance noise may be a significant cause of handpiece noise and it would be meaningful that this study can provide clues for the analysis of the causes of handpiece noise which has been scarcely studied so far. Further comprehensive studies shall be required to explore the whole mechanism of dental handpiece noise generation.

## Conclusion

Vibroacoustic analysis of the air layer contained within the dental air turbine handpiece showed the resonance peak noise at 4478.92 Hz under the simulated conditions.
